# Defining the criteria for identifying constitutional epimutations

**DOI:** 10.1186/s13148-016-0207-4

**Published:** 2016-04-18

**Authors:** Mathew A. Sloane, Robyn L. Ward, Luke B. Hesson

**Affiliations:** Adult Cancer Program, Lowy Cancer Research Centre and Prince of Wales Clinical School, UNSW Australia, Kensington, Sydney, New South Wales 2052 Australia; The University of Queensland, Level 3 Brian Wilson Chancellery, Brisbane, Queensland 4072 Australia

**Keywords:** Constitutional epimutation, RB1, Retinoblastoma, Methylation

## Abstract

In the January 2016 issue of *Clinical Epigenetics*, Quiñonez-Silva et al. (Clin Epigenetics 8:1, 2016) described a possible constitutional epimutation of the *RB1* gene as a cause of hereditary predisposition to retinoblastoma. The term constitutional epimutation describes an epigenetic aberration in normal tissues that predisposes to disease. The data presented by Quiñonez-Silva et al. are interesting, but further analysis is required to demonstrate a constitutional epimutation in this family. Here, we define the criteria and describe the experimental approach necessary to identify an epigenetic aberration as a constitutional epimutation.

## Basic criteria that define constitutional epimutations

An epimutation describes an epigenetic aberration that results in the transcriptional silencing of a gene that is normally active or the expression of a gene that is normally inactive [1]. Epimutations that are widely distributed in normal tissues and predispose to disease are known as constitutional epimutations [2]. Epigenetic silencing may occur throughout all normal tissues (soma-wide) or show mosaicism depending on the mechanistic basis of the epimutation. Constitutional epimutations have been described in various genes and diseases including Lynch syndrome (*MLH1* and *MSH2*) [3, 4], fragile X syndrome (*FMR1*) [5], α-thalassaemia (*HBA2*) [6] and several imprinting disorders [1]. Constitutional epimutations are confined to one allele and may occur in the absence or presence of an in-*cis* genetic variant and termed primary or secondary epimutations, respectively. Co-segregation of an epimutation with disease provides very strong evidence that it plays a role in disease causality; however, this is not a defining characteristic due to their potential reversal in the germ line and consequent non-Mendelian pattern of inheritance [3].

## Experimental approach for identifying constitutional epimutations

To define an epigenetic aberration as a constitutional epimutation, the following must be demonstrated. Firstly, promoter DNA hypermethylation must be confined to one allele in normal tissues derived from multiple germ layers. Peripheral blood, hair follicles and saliva or buccal mucosa are representative derivatives of the mesoderm, ectoderm and endoderm, respectively, which are routinely assayed in constitutional epimutation studies. Secondly, validation of the presence and level of methylation should be performed using at least two independent methods, such as bisulphite pyrosequencing and clonal bisulphite sequencing, to reliably eliminate confounders including cloning bias and incomplete bisulphite conversion. Bisulphite pyrosequencing can quantify the average level of methylation across both alleles [7], whereas clonal bisulphite sequencing across an informative (heterozygous) promoter variant can confirm whether methylation is allele-specific. Thirdly, the methylated allele must show partial or complete transcriptional silencing. Allele-specific mRNA expression levels can be accurately quantified with cDNA pyrosequencing [8] using an informative expressed variant. This usually requires extensive knowledge of the locus through variant haplotyping in family members. Finally, there must be co-segregation of the methylated and transcriptionally silent allele with disease.

The study by Quiñonez-Silva et al. [9] has not definitively demonstrated a constitutional epimutation of the retinoblastoma 1 (*RB1*) gene in this family. Firstly, germ line genetic testing was not performed meaning a genetic cause of retinoblastoma cannot be ruled out. This would normally be considered a high priority especially in light of the strong paternal history of retinoblastoma. On the other hand, the mother of the index case, and the origin of the apparent epimutation, had no personal or family history of retinoblastoma. Secondly, the authors tested constitutional methylation of the RB1 promoter in only one germ layer (mesoderm, peripheral blood) and with only one method (clonal bisulphite sequencing). It is therefore unclear whether methylation is widely distributed in normal cells or whether the striking pattern of methylation is reproducible using an independent method. Methylation in melanoma cells from the index case may represent a ‘second somatic hit’ and is not necessarily evidence of a constitutional epimutation. Thirdly, this case is complicated by the observation of hypermethylation on both the maternal variant (c.[-187G; -188G]) allele and the paternal wild-type (c.[-187T; -188T]) allele possibly related to the fact that *RB1* is imprinted [10]. Fourthly, critical data is missing from the manuscript. For example, methylation of the *RB1* promoter was reported in multiple family members across two generations but no data were provided to support this. Fifthly, segregation analysis of a panel of microsatellite markers spread throughout the genome was described, but this provided no insight into the inheritance pattern of the epimutation as proposed. Sixthly, allele-specific *RB1* expression in normal tissue was not investigated. Expression and methylation analyses across multiple tissues would have shown whether the maternally inherited *RB1* allele showed constitutional loss of expression and would have clarified the relationship between transcriptional activity and methylation levels at the promoter. Germ line genetic testing may have provided informative expressed variants to enable this. For these reasons, it remains to be determined whether this family displays a bona fide constitutional epimutation. Progress in identifying disease predisposition genes offers the potential to identify novel constitutional epimutations. Candidate genes with suspected constitutional epimutations, including *RB1*, should be investigated using the above criteria as a guide.

## Abbreviations

*RB1*: retinoblastoma 1

## References

[1] Hesson LB, Hitchins MP, Ward RL (2010). Epimutations and cancer predisposition: importance and mechanisms. Curr Opin Genet Dev.

[2] Hitchins MP (2015). Constitutional epimutation as a mechanism for cancer causality and heritability?. Nat Rev Cancer.

[3] Hitchins MP, Wong JJ, Suthers G, Suter CM, Martin DI, Hawkins NJ (2007). Inheritance of a cancer-associated MLH1 germ-line epimutation. N Engl J Med.

[4] Ligtenberg MJ, Kuiper RP, Chan TL, Goossens M, Hebeda KM, Voorendt M (2009). Heritable somatic methylation and inactivation of MSH2 in families with Lynch syndrome due to deletion of the 3′ exons of TACSTD1. Nat Genet.

[5] Oberle I, Rousseau F, Heitz D, Kretz C, Devys D, Hanauer A (1991). Instability of a 550-base pair DNA segment and abnormal methylation in fragile X syndrome. Science.

[6] Tufarelli C, Stanley JA, Garrick D, Sharpe JA, Ayyub H, Wood WG (2003). Transcription of antisense RNA leading to gene silencing and methylation as a novel cause of human genetic disease. Nat Genet.

[7] Tost J, Gut IG (2007). DNA methylation analysis by pyrosequencing. Nat Protoc.

[8] Ronaghi M, Uhlen M, Nyren P (1998). A sequencing method based on real-time pyrophosphate. Science.

[9] Quiñonez-Silva G, Davalos-Salas M, Recillas-Targa F, Ostrosky-Wegman P, Aranda DA, Benitez-Bribiesca L (2016). Monoallelic germline methylation and sequence variant in the promoter of the RB1 gene: a possible constitutive epimutation in hereditary retinoblastoma. Clin Epigenetics.

[10] Kanber D, Berulava T, Ammerpohl O, Mitter D, Richter J, Siebert R (2009). The human retinoblastoma gene is imprinted. PLoS Genet.

